# A counterweight model for understanding and treating persecutory delusions

**DOI:** 10.1017/S0033291725001242

**Published:** 2025-05-13

**Authors:** Daniel Freeman, Louise Isham, Felicity Waite

**Affiliations:** 1 Department of Experimental Psychology, University of Oxford, Oxford, UK; 2 Oxford Health NHS Foundation Trust, Oxford, UK

**Keywords:** Delusions, Persecutory, Paranoia, Cognitive, Treatment, Schizophrenia, Psychosis

## Abstract

Direct challenge seldom leads to change in strongly held beliefs such as persecutory delusions. A better route is to develop an alternative belief that can coexist with the delusion. The best such beliefs function as counterweights to the delusion. Over time, the scales shift. The alternative belief becomes more powerful than the delusion. In this paper, we set out such a model of persecutory delusions (or severe paranoia) and describe how it inherently translates theoretical understanding into treatment routes. Severe paranoia occurs when the adaptive cognitive processes of deciding whether to trust become overly weighted to mistrust. An inaccurate threat belief is formed, and the person feels very unsafe. Hence, overcoming the delusion means developing a counterweighting belief. It means building the alternative view that the world is safe enough for the person now and going forward. This, in turn, is done by *experiencing* safety. However, the pull of paranoia is strong due to multiple factors such as past history, anxious arousal, hallucinations, feelings of vulnerability, use of defenses, withdrawal, worry, difficulties distancing from fears, and a sense of defeat. These factors can prevent the person from feeling safe in even the most benign environments. Therefore, counterweights must be developed for these factors. For instance, feeling vulnerable can be counterweighted by developing self-confidence. Excessive time spent worrying can be counterweighted by devoting more time to thinking about meaningful activities. The counterweight approach provides a non-confrontational, empathic, personalized way to lift the burden of paranoia from a patient with persecutory delusions.

*I think my light bulb moment was realising that a lot of obviously the reasons I felt unsafe was due to my past. I used to describe it as like a pit. This darkness all around me. Finding that light and opening doors to other people and actually letting people in to help you to then realise that you are actually safe and other people can make you feel safe, and you actually can feel safe in yourself as well, made me then start to have more lights, and then that pit got smaller and then eventually it was like a pothole, which I could just step out of*. Rachel.

## Introduction

Rachel believed that people wanted to hurt her, that everyone walking past her house was a potential danger, and sometimes that people were setting fire to her home. She mistrusted most people. Over the course of the Feeling Safe program (Freeman et al., 2021) Rachel realized that her past trauma was a key driver of her fears. An alternative view was raised: that perhaps people could be trusted now. Over many months, Rachel practiced trusting people. She learned that she was safe. Her paranoia faded away. In other words, Rachel developed a sense of safety that functioned as a counterweight to her fears. Over time, the scales tipped and safety – rather than fear – became her dominant belief. Persecutory delusions, such as those experienced by Rachel are one of the most common difficulties in psychosis presentations (Lemonde et al., 2021; Collin, Rowse, Martinez, & Bentall, 2023; Pappa et al., 2025) but are all too often resistant to standard treatments. In this paper, we describe a way of understanding paranoia, of talking about it with patients – and of overcoming it.

### What are persecutory delusions?

Persecutory delusions are strongly held, but incorrect, beliefs that others are deliberately trying to harm the person. That harm may, for instance, be psychological, social, financial, or physical. Such beliefs can produce extremely negative consequences. The attempt to deal with the perceived threat – for example, by withdrawing – can be highly disruptive to everyday life. Negative affect, especially anxiety and depression, but sometimes also anger, is common. Life can feel like a battle. Often, persecutory delusions are an extension of delusions of reference, in which people believe that others are talking about them behind their back or that messages are being sent to them, or of hallucinatory experiences such as hearing voices.

We conceptualize persecutory delusions as inaccurate threat beliefs that are attempts to make sense of events (Freeman et al., 2002). They are the extreme end of a spectrum of paranoia in the general population. Many people have a few paranoid thoughts, and a few people have many paranoid thoughts (Bebbington et al., 2013; Freeman et al., 2005; Neidhart, Mohnke, Vogel, & Walter, 2024). This is unsurprising. Decisions about whether to trust or mistrust other people are an inescapable part of human cognition – after all, real dangers exist, people do bad things to others, and safety can seldom be completely guaranteed. Paranoia is what results when that decision-making skews excessively to the negative so that judgments are inaccurate (Freeman, 2016). In other words, it arises out of everyday risk estimation gone awry. Paranoia occurs transdiagnostically (e.g. Alsawy, Wood, Taylor, & Morrison, 2015; D’Agostino, Monti, & Starcevic, 2019; Varghese et al., 2011). In conditions such as anxiety and depression it has been found – most likely due to the negative effects on interpersonal relationships – to be a marker of poorer outcomes (Bird et al., 2021; Wiedemann et al., 2024). Of course, paranoia can be an understandable reaction to events. Patients are more likely to live in difficult settings and experience hostility. A person can both have paranoia and face genuine threats. But ‘the dose makes the poison’. Too much paranoia – a high concentration – can create damaging effects. In persecutory delusions, the estimation of danger has become so dominant that the person feels extremely, and often debilitatingly, unsafe.

### The weight of paranoia

*I couldn’t not believe my beliefs. I was convinced about them…and because my beliefs became so loud and just dominated me, they weren’t helping me at all.* Steve.

Why do persecutory beliefs become such a persuasive way of understanding events? Why does their voice drown out other perspectives? Our view is that they carry such weight because so many factors contribute to their existence and maintenance – though the number and relative influence of those factors will vary from person to person. This complexity is illustrated in our study of 22 cognitive and social causes of paranoia (Freeman & Loe, 2023). We found that all 22 causes were individually associated with paranoia, and in a combined model, 13 factors explained two thirds of the variance in paranoia. The 13 factors were: within-situation defense behaviors, negative images, negative self-beliefs, discrimination, dissociation, aberrant salience, anxiety sensitivity, agoraphobic distress, worry, less social support, agoraphobic avoidance, less analytical reasoning, and alcohol use. Another illustration is the wide range of other factors researchers are examining to understand paranoia, including aberrant belief updating (Sheffield, Suthaharan, Leptourgos, & Corlett, 2022; Barnby, Mehta, & Moutoussis, 2022; Rossi-Goldthorpe et al., 2024), social group threat detection (Raihani & Bell, 2019), amplified threat processing and impaired emotion regulation (Lincoln, Sundag, Schlier, & Karow, 2018; Walther et al., 2022), social isolation (Contreras et al., 2022; Fett et al., 2022), early life adversity (Bentall, Wickham, Shevlin, & Varese, 2012), PTSD symptoms (Hardy et al., 2021; Panayi et al., 2024), and attachment style (MacBeth, Schwannauer, & Gumley, 2008; Sood, Carnelley, & Newman‐Taylor, 2022). At a neurobiological level of explanation, overactivation and hyperconnectivity of the amygdala – reflecting amplified detection of salience and threat and insufficient top-down regulation – have been repeatedly linked to paranoia (Pinkham et al., 2015; Fan et al., 2021; Pinkham et al., 2022; Walther et al., 2022).

**Figure 1.**
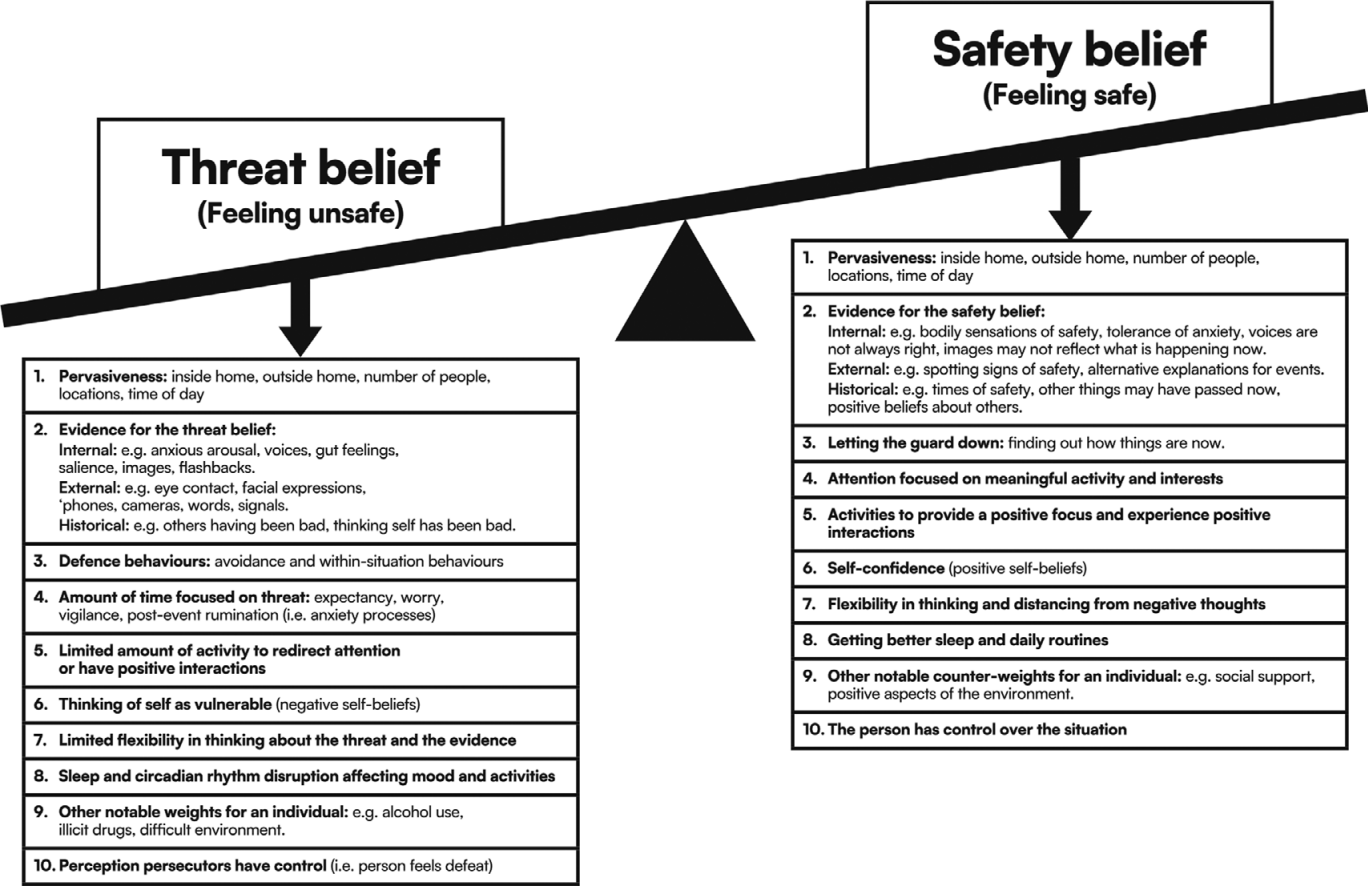
A counterweight model of paranoia.

In our counterweight model of paranoia, we list 10 categories of factors that provide weight to the inaccurate threat belief (see Figure 1 and online Supplementary materials Figure S1). The focus is on factors that are tractable to intervention and that patients want treated (Freeman, Taylor, Molodynski, & Waite, 2019). The factors are designed to accommodate the influence of past experience on current processing (e.g., trauma may have had effects on, for example, self-beliefs, beliefs about others, anxious arousal, and imagery). The model is designed to provide structure to a clinical assessment of persecutory delusions and to do so from the patient’s perspective. The conversation in that assessment starts at the belief itself. We discuss how much feeling unsafe pervades the person’s life, and the types of direct evidence supporting that feeling. Often, the person’s threat belief is fueled by the physiological effects of anxiety. The anxiety can sometimes be accompanied by negative images (Kingston et al., 2025; Morrison et al., 2002). Sometimes the person hears a voice telling them that they are vulnerable and will be attacked, which is then believed (Sheaves et al., 2023). The long shadow of negative past events is often perceptible. Such events may involve things others have done to the person or actions the person feels guilty about. We then move on to a discussion of the defense behaviors – safety-seeking behaviors (Salkovskis, 1991) – that the person adopts to reduce the likelihood of harm. Often, the person avoids situations they find threatening. If they do enter such situations, they use subtle strategies for protection (e.g., rushing, choosing a quiet time of day, making themselves inconspicuous). Next, we assess how much the person is anticipating and thinking about threat, and, conversely, the time they spend in positive activities, such as social contact. Most patients devote considerable time to worrying about the threats and much less to other activities. We then broaden out into a discussion of how the person may feel inherently vulnerable in regards to other people due to negative self-beliefs (Collett, Pugh, Waite, & Freeman, 2016; Humphrey et al., 2021; Waite et al., 2023). We may note that it is hard for the person to view events in different ways, and that the situation is not helped by disruption to sleep and routines (Freeman & Waite, 2025). Typically, we conclude by exploring how much control the person feels the persecutors possess. This signposts that we want the person themselves to have greater control. In sum, the process we follow in the assessment allows us to build a personalized picture of the individual’s situation. It gives us a tailored appraisal of the weights pulling the person’s understanding towards paranoia. Descriptions of the mechanistic links between the factors and paranoia are provided in Table 1.

**Table 1.** Examples of mechanistic pathways to severe paranoia

Factor	Example mechanistic explanations
Pervasiveness	The greater the number of potential locations and periods of time that attacks could take place then the greater the number of triggers of anxious feelings, worry, and paranoid thoughts.
Evidence for the threat belief	These are taken as the most proximal indications of current threat: Anxious arousal indicates to the person that there is danger. Voices may tell the person that they are vulnerable and that they will be attacked. An image may show the person what will happen to them. Eye contact is taken that other people know who they are and may attack. People may be telling others about them on their mobile phones. Others have attacked them in the past and hence may do so now. The person will be punished for things they have done in the past.
Defense behaviors	The person may avoid situations and therefore fail to receive corrective information about safety. In situations of threat, the person carries out actions to keep themselves safe. The absence of harm is attributed to the use of the defenses. Defenses end up protecting the fears and preventing the person from having opportunities to learn that things may have changed.
Amount of time focused on threat	The person is anticipating a threat. Worry elaborates the fears, keeps them in mind, and leads to over-estimation of the likelihood of harm. Worry can also turn benign events into potentially threatening ones.
Limited amount of activity	The person is often inactive, and therefore mood may be lowered, and the mind is unoccupied and turns to fears. There are fewer experiences of safety around other people.
Negative self-beliefs	Negative self-beliefs lead the person to feel odd, different, and inferior to other people. This puts the person in a position of vulnerability in relation to other people. Paranoia builds on feelings of vulnerability.
Limited flexibility in thinking about the threat and the evidence	The failure to have alternative explanations means that there are no real competitors to the fears, and therefore, they assume dominance. Having only one main explanation of events means there is less reason to doubt it, pause, and gain psychological distance from it.
Sleep and circadian rhythm disruption	Disrupted sleep increases anxiety and the expectation of threat. It also increases the occurrence of internal anomalies of experience that are taken as a sign that something is wrong.
Other notable weights	Illicit drugs can directly trigger anxiety, worry, and anomalies of experience that feed into the occurrence of paranoia. Real threats in the environment make it harder to feel safe and can provide further justification to not trust other people.
Perception persecutors have control	The person thinks that they cannot control the situation, and feels defeated (often accompanied by a low mood), which means it is harder to think differently or re-engage in activities that could ease fears.

### Overcoming paranoia: counterweights

*Nobody can tell you, you’ve got to learn yourself. I found out a) it was okay b) there was life outside my house that I wanted to be part of and c) people smiled.* Gill.

Many patients lack alternative explanations for the delusion and its associated evidence. If they do have alternative ideas, these may be unpalatable and limited in explanatory power (e.g., ‘mental illness’, ‘brain damage’) (Freeman et al., 2004). We therefore provide a series of counterweighting beliefs or behaviors that are positive; can coexist with the person’s existing position; and if adopted will inherently tip the scales away from the persecutory fears. The primary counterweight is a belief that the person may be safe *now.* This is a position that does not dispute the past. And the counterweight can be even more circumscribed: that the person is safe enough now in certain places at certain times (to do things that they would like to do). Of course, absolute safety cannot be guaranteed for anyone, and certain environments at certain times can be dangerous. The development of safety is best learned from direct experience. Within an inhibitory learning framework of understanding (Craske et al., 2014), the new learning of safety will constrain and dampen the old learning that others intend harm. Relatedly, the counterweight approach has similarities to a retrieval competition account of cognitive behavior therapy for emotional disorders, in which the principal mechanism of action of intervention is considered as ‘strengthening competitor representations in memory that are positive rather than negative in valence’ (Brewin, 2006). It is also consistent with the general reasoning literature: considering an alternative, accessible, and plausible explanation is more likely to debias judgements (Hirt & Markman, 1995; Wharton, Cheng, & Wickens, 1993; Sanna, Schwarz, & Stocker, 2002; Hirt, Kardes, & Markman, 2004).

However, relearning safety can be extremely difficult when so many weights still pull in favor of the persecutory delusion. These weights can make even benign situations feel unsafe (e.g., ‘If I had not left early, I would have been harmed’, ‘I had an image of the harm they were going to do to me’, ‘Later I realized that the smile was because they were up to something’). Therefore, at least some of these weights must be lightened. Our framework for the introduction of counterweights is provided in Table 2. It is important to convey to the person that the existence of the particular weight is understandable – that it makes sense, for example, given their life experiences or as a response to their fears. But that forces a heavy burden upon them. A positive counterweight can then be introduced, and its potential benefits described. The merit of this approach is that many routes for treatment become evident. For example, increasing activities, sleeping better, building up self-confidence, looking for signs of safety, letting down one’s guard to find out how the world is now, learning to tolerate anxious feelings, letting oneself rather than the voices decide what is going on, and getting some distance from troubling thoughts. It is notable that the approach intrinsically introduces flexibility into thinking about difficulties because of the introduction of counterweights.

**Table 2.** Examples of how to pivot with patients to a counterweight

The weight	Stating the problem (with understanding why it occurred)	The problem with the problem	New counterweight position	The benefits of the counterweight position
1. Persecutory delusion	‘Given what has happened to you in the past, it is unsurprising that you are very wary and fearful of other people.’	‘The trouble is that it makes you anxious, wears you down, and stops you doing the things you want to do.’	‘Perhaps things have changed now, and there are some situations, places, or times that are safe enough for you to do what you want.’	‘This could take the weight of anxiety off your shoulders and help you take back control and do what you want to do.’
2. Worry	‘Many people who feel unsafe worry a lot. And they generally worry because they think it’ll help. Worry seems like a way of being on guard. Of preventing danger. Of staying safe.’	‘It is a very understandable reaction. But it may not be the most helpful. It means our fears play on our minds, sometimes for hours on end, day after day. It can feel exhausting.’	‘Maybe it is time to keep worry to a short period. For you to get back your headspace to think about the things you want to think about.’	‘The time you used to spend worrying could be filled with activities that are enjoyable and more productive.’
3. Negative voices	‘The voices are saying bad things are going to happen, and that is not easy to ignore at all.’	‘It makes the world feel very frightening.’	‘But perhaps you can decide what is happening.’	‘This will put you in charge of decision-making.’
4. Anxious arousal	‘Your body is telling you there is danger from other people. It is your internal alarm going off. That is not surprising given what has happened to you.’	‘The fears about what it means lead you to stop doing things. The feelings of anxiety are in charge.’	‘But perhaps it is just anxiety. A shadow from the past. Maybe it is not a sign now of anything more than that you are anxious. And that really the threat has stopped. The alarm may just be a nuisance noise. Maybe there is a chance to develop greater calmness instead.’	‘You may well get used to those anxious feelings, let them go, and get on with the things you want to do.’
5. Defenses	‘It is completely natural that you have put up the defenses – avoiding situations in which you feel you will be attacked.’	‘The problem is that you miss out on doing so many things you used to do. And it can be exhausting being on guard so much of the time.’	‘It is true that there are risks in the world. But to live our lives, we need to find enough safety to do ordinary activities. We sometimes need to lower our guard and take a look. You may gain confidence that you can safely do the things you want.’	‘It could be that you have more freedom and control now than you think.’

The model can be used to formulate a person’s difficulties with paranoia. Early in the intervention, we want to understand the weights pulling the person towards the delusion. But we then shift to identify and build up counterweights. A person with severe paranoia will typically need to follow several treatment routes, one at a time. But the counterweight approach typically identifies many options, allowing patient choice to be built into treatment provision. This approach generally produces a range of positive outcomes (Freeman et al., 2021; Jenner et al., 2024). For some people, the weight of paranoia is lifted a little. For others, the change is enough to balance the scales. And sometimes the scales shift so dramatically in favor of safety that the burden of paranoia is lifted entirely.

### Models are indispensable

A suitable model is essential for powerful psychological intervention. It allows us to identify treatment targets, share understanding with patients, and guide the focus of sessions. The counterweight model we describe here was developed to guide both psychological understanding and treatment, and underlies the face-to-face Feeling Safe program (Freeman et al., 2016) and the guided online program Feeling Safer (Freeman et al., in revision), both of which have shown strong treatment effects. For example, in a randomized controlled trial, Feeling Safe produced a large further reduction in persecutory delusions above an alternative psychological intervention delivered by the same therapists (Freeman et al., 2021). Indeed, the principal counterweight (a feeling of safety) is signaled from the outset by the name of these programs. But an appropriate model alone is insufficient. Successful treatment requires other elements too. Elsewhere, we have described 10 key principles, including the use of counterweights, for developing psychological treatment for psychosis (Freeman, 2024). These include respect for patients, contributions from people with lived experience, and precise and rigorous treatment delivery. We also highlight the fundamental importance of collecting outcomes at each intervention session. We must be able to gauge whether a mechanistic target is being successfully addressed and whether there is improvement in the persecutory delusion. There is still work to be done on developing a comprehensive, patient-focused set of assessments to guide paranoia research and treatment. As we seek to improve the treatment of persecutory delusions, identifying further significant and tractable intervention weights and counterweights, that set of assessments must evolve accordingly.

## Supporting information

Freeman et al. supplementary material Freeman et al. supplementary material

## Supplementary material

The supplementary material for this article can be found at http://doi.org/10.1017/S0033291725001242.
