# Supplementary material for: A counterweight model for understanding and treating persecutory delusions
Source: Psychol Med. 2025 May 13;55:e141. doi: 10.1017/S0033291725001242 (PMC12094649; doi:10.1017/S0033291725001242)
Supplement: Freeman et al. supplementary material [file S0033291725001242sup001.docx]

| **1. Pervasiveness:** inside home, outside home, number of people, locations, time of day |
| --- |
| **2. Evidence for the threat belief:** **Internal:** e.g., anxious arousal, voices, gut feelings, salience, images, flashbacks.  **External:** e.g., eye contact, facial expressions, ‘phones, cameras, words, signals. **Historical:** e.g., others having been bad, thinking self has been bad. |
| **3. Defence behaviours:** avoidance and within-situation behaviours |
| **4. Amount of time focused on threat:** expectancy, worry, vigilance, post-event rumination (i.e. anxiety processes) |
| **5. Limited amount of activity to redirect attention or have positive interactions** |
| **6. Thinking of self as vulnerable** (negative self-beliefs) |
| **7. Limited flexibility in thinking about the threat and the evidence** |
| **8. Sleep and circadian rhythm disruption affecting mood and activities** |
| **9. Other notable weights for an individual:** e.g., alcohol use, illicit drugs, difficult environment. |
| **10. Perception persecutors have control** (i.e., person feels defeat) |

| **1. Pervasiveness:** inside home, outside home, number of people, locations, time of day |
| --- |
| **2. Evidence for the safety belief:** **Internal:** e.g., bodily sensations of safety, tolerance of anxiety, voices are not always right, images may not reflect what is happening now. **External:** e.g. spotting signs of safety, alternative explanations for events. **Historical:** e.g. times of safety, other things may have passed now, positive beliefs about others. |
| **3. Letting the guard down:** finding out how things are now. |
| **4. Attention focused on meaningful activity and interests.** |
| **5. Activities to provide a positive focus and experience positive interactions** |
| **6. Self-confidence** (positive self-beliefs) |
| **7. Flexibility in thinking and distancing from negative thoughts.** |
| **8. Getting better sleep and daily routines.** |
| **9. Other notable counter-weights for an individual:** e.g. social support,  positive aspects of the environment. |
| **10. The person has control over the situation** |

**Safety belief**

**Threat belief**
